# Quantitative bile acid profiling in healthy adult dogs and pups from serum, plasma, urine, and feces using LC-MS/MS

**DOI:** 10.3389/fvets.2024.1380920

**Published:** 2024-06-14

**Authors:** Emre Karakus, Anna-Lena Proksch, Andreas Moritz, Joachim Geyer

**Affiliations:** ^1^Institute of Pharmacology and Toxicology, Faculty of Veterinary Medicine, Justus Liebig University Giessen, Giessen, Germany; ^2^Clinic of Small Animals—Internal Medicine, Faculty of Veterinary Medicine, Justus Liebig University Giessen, Giessen, Germany

**Keywords:** bile acids, dog, pup, plasma, serum, urine, feces, LC-MS/MS

## Abstract

Synthesis and secretion of bile acids (BA) is a key physiological function of the liver. In pathological conditions like portosystemic shunt, hepatic insufficiency, hepatitis, or cirrhosis BA metabolism and secretion are disturbed. Quantification of total serum BA is an established diagnostic method to assess the general liver function and allows early detection of abnormalities, liver disease progression and guidance of treatment decisions. To date, data on comparative BA profiles in dogs are limited. However, BA profiles might be even better diagnostic parameters than total BA concentrations. On this background, the present study analyzed and compared individual BA profiles in serum, plasma, urine, and feces of 10 healthy pups and 40 adult healthy dogs using ultra-high performance liquid chromatography coupled to electrospray ionization mass spectrometry. Sample preparation was performed by solid-phase extraction for serum, plasma, and urine samples or by protein precipitation with methanol for the feces samples. For each dog, 22 different BA, including unconjugated BA and their glycine and taurine conjugates, were analyzed. In general, there was a great interindividual variation for the concentrations of single BA, mostly exemplified by the fact that cholic acid (CA) was by far the most prominent BA in blood and urine samples of some of the dogs (adults and pups), while in others, CA was under the detection limit. There were no significant age-related differences in the BA profiles, but pups showed generally lower absolute BA concentrations in serum, plasma, and urine. Taurine-conjugated BA were predominant in the serum and plasma of both pups (68%) and adults (74–75%), while unconjugated BA were predominant in the urine and feces of pups (64 and 95%, respectively) and adults (68 and 99%, respectively). The primary BA chenodeoxycholic acid and taurocholic acid and the secondary BA deoxycholic acid and lithocholic acid were the most robust analytes for potential diagnostic purpose. In conclusion, this study reports simultaneous BA profiling in dog serum, plasma, urine, and feces and provides valuable diagnostic data for subsequent clinical studies in dogs with different kinds of liver diseases.

## 1 Introduction

**Figure 1:**
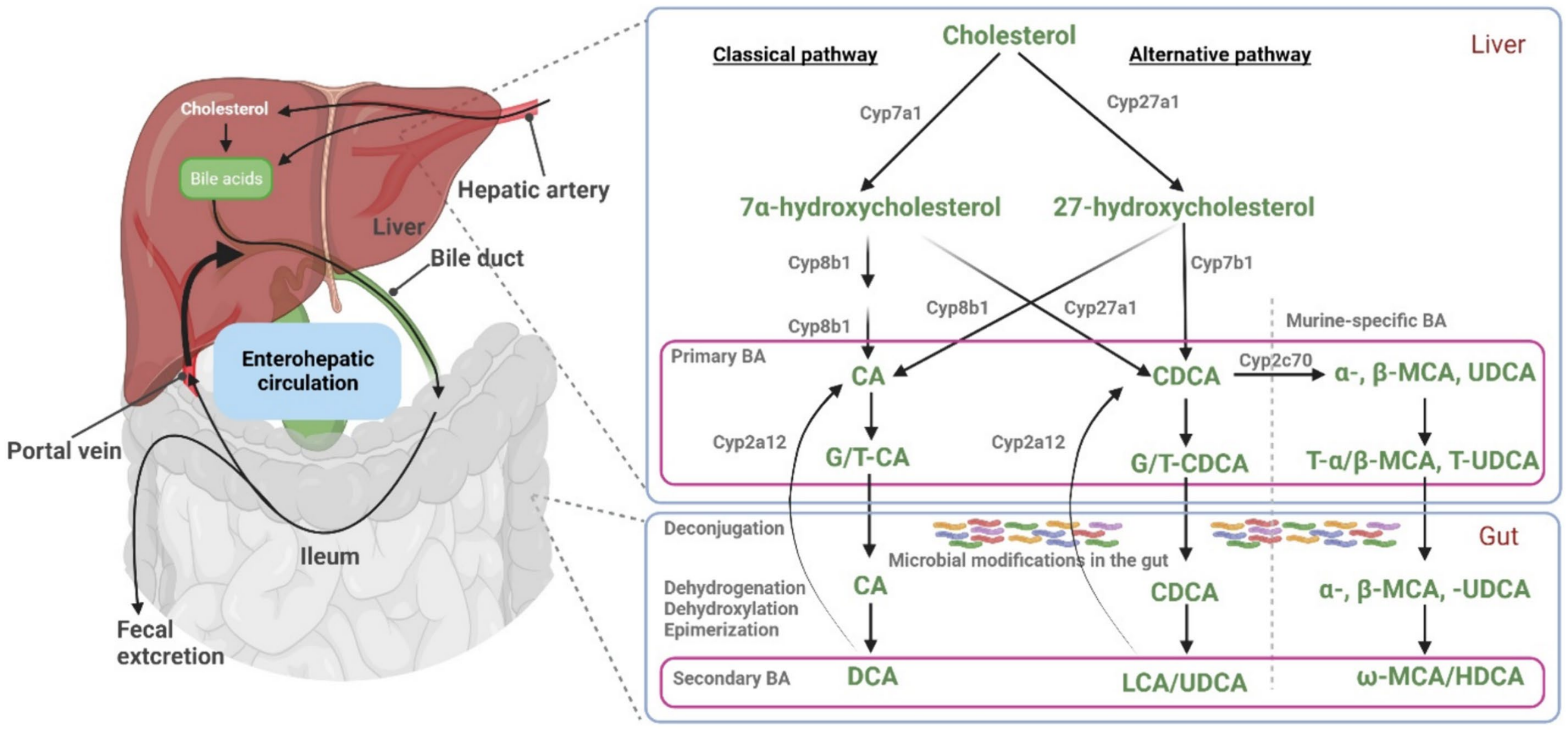
Bile acid synthesis, metabolism, and enterohepatic circulation. Key enzymes involved in BA synthesis and metabolism are illustrated. In hepatocytes, primary BA (CA, CDCA, and α/β-MCA) are synthesized and then conjugated with taurine and glycine (T/G), before they are excreted into the intestine via bile. Within the gut, primary BA undergo microbial modifications, resulting in the formation of secondary BA. At least in mice, liver Cyp2a12 can convert these secondary BA to primary BA. Under normal physiological conditions, a fraction of BA bypasses the first-pass hepatic clearance and enters the systemic circulation. Part of this blood BA pool is filtered by the renal glomeruli and excreted via the urine. Figure modified from Wahlström et al. (10) and Honda et al. (11). Figure created with BioRender.

Bile acids (BA) are a class of amphipathic molecules characterized by a hydroxylated steroid nucleus and a carboxylic acid terminal group. *De novo* BA biosynthesis occurs in the liver and ends up with the primary BA cholic acid (CA) and chenodeoxycholic acid (CDCA). Both are formed from cholesterol in the liver, involving numerous enzymatic transformations (1). In a second step, these primary BA are conjugated with taurine or glycine to form glyco-CA, glyco-CDCA, tauro-CA, and tauro-CDCA, respectively. Notably, the conjugation pattern varies among species, with glycine conjugation being predominant in humans, minipigs, and hamsters, while BA amidation with taurine is prevalent in mice, rats, and dogs (2, 3). In mice, CDCA can also be converted to muricholic acid (MCA) and ursodeoxycholic acid (UDCA), making mouse bile more hydrophilic than human bile (4). BA are secreted from hepatocytes into bile via the canalicular bile salt export pump (BSEP) and then are stored in the gallbladder in most species (5). After release of BA into the intestine, most of the conjugated BA are reabsorbed in the terminal ileum via the apical sodium-dependent bile acid transporter (ASBT) that is expressed at the brush border membrane of ileal enterocytes (6). However, a certain proportion of the intestinal BA escapes absorption and these BA finally enter the colon. Here, the resident gut microbiota promotes the deconjugation and biotransformation of primary to secondary BA such as deoxycholic acid (DCA), lithocholic acid (LCA), or UDCA (7–9) (Figure 1). Accordingly, most fecal BA are deconjugated. While a small amount (~5%) of BA are ultimately eliminated from the body via feces, the remaining BA are passively reabsorbed from the colon (12). These BA then return to the liver via the portal vein and are largely reabsorbed into hepatocytes via the Na^+^/taurocholate cotransporting polypeptide (NTCP) or transporters from the organic anion transporting polypeptide (OATP) carrier family (13). Within hepatocytes, primary and secondary BA then are (re)-conjugated with taurine or glycine.

In addition to their well-known physiological role (e.g., for the elimination of cholesterol, and the absorption of dietary lipids and fat-soluble vitamins), BA are important regulators of various signaling pathways (e.g., involving JNK1/2, AKT, and ERK1/2) crucial for BA metabolic and immune-related functions (14). BA also interact with pivotal receptors, including the nuclear receptors farnesoid X receptor (FXR), pregnane X receptor (PXR), and vitamin D receptor, as well as the membranous G protein-coupled receptor TGR5 (12, 15). The extensive interaction of these receptors emphasizes the role of BA as master regulators of different complex physiological processes (16). Activation of FXR in the gut results in the secretion of human fibroblast growth factor 19 (FGF-19) into the portal blood circulation that finally exerts regulatory effects on glucose and lipid metabolism (17). On the other hand, elevated BA concentrations can exert cytotoxic and even carcinogenic effects on cells (18, 19). Consequently, in a healthy liver, the uptake, synthesis, and release of BAs are precisely regulated to maintain the desired concentration during their enterohepatic circulation (20). However, pathological conditions, such as cholestatic liver disorders (21) and portosystemic shunt (22), can cause toxic BA accumulation within the liver and dramatically elevated BA in the systemic circulation.

In recent years, the number of BA profiling and quantification studies has increased significantly, with a strong emphasis on quantitative BA profiles across a wide range of biological samples. The in-depth study of BA profiles in humans has enabled their use as non-invasive diagnostic or prognostic markers for numerous hepatobiliary diseases, including (but not limited to) non-alcoholic fatty liver disease, non-alcoholic steatohepatitis, cholangiocarcinoma, hepatitis B and C virus (HBV/HCV) infections, alcoholic liver disease, primary biliary cirrhosis, primary sclerosing cholangitis, or autoimmune hepatitis (23–25). In addition, the analysis of the BA profiles offers a valuable approach to investigate potential regulatory functions of BA via different signaling pathways (26).

To date, numerous scientific studies have addressed the comprehensive profiling of BA in both human subjects and preclinical animal models (3). For instance, Bathena et al. (27) comprehensively described the BA composition in human serum and urine, while John et al. (28) performed an extensive analysis of BA in mouse plasma, urine, gallbladder, liver, feces, and adipose tissue. Sangaraju et al. (29) quantitatively analyzed 50 different BA in various matrices (including plasma and urine) of different species (human, monkey, rabbit, dog, and rat). Additionally, Thakare et al. (30) expanded the cross-species BA analysis up to eleven different species, also including beagle dogs. In most of these studies involving dogs, BA analysis was either presented individually across various matrices or as collective report combining several matrices, and most of these studies were performed only in mixed breeds or beagle dogs (17, 31–36). However, the lack of BA profiling studies in different age groups, the low number of studies for healthy clinical patient collectives of very different dog breeds, and the poorly characterized dietary effects on the BA profile still limit our understanding of the physiological range in BA concentrations and abnormal values in disease conditions.

Based on this, the analysis of canine BA profiles is of great importance in veterinary medicine and research due to its relevance in elucidating various physiological and pathophysiological aspects of canine health. In particular, the analysis of serum BA in canine populations provides valuable insights into the diagnosis and monitoring of liver diseases, such as hepatitis, cholestasis, portosystemic shunt, extrahepatic bile duct obstruction, and hepatic neoplasia which are common in dogs (37). In addition, understanding the variation in BA profiles across different age groups of dogs is essential, as it may provide insights into age-related changes in metabolism and gastrointestinal health.

In the present study, we analyzed the profiles of 22 individual BA in serum, plasma, urine, and feces samples from 10 healthy pups and from 40 adult healthy dogs using ultra-high performance liquid chromatography (UHPLC) coupled to electrospray ionization mass spectrometry (ESI-MS/MS). To our knowledge, this is the first report that compares the composition of BA in adult dogs and pups simultaneously across different matrices, including a direct comparison of BA concentrations in plasma and serum samples from each dog.

## 2 Material and method

### 2.1 Chemicals

All chemicals and solvents were of the highest purity commercially available. BA standards ω-muricholic acid (ω-MCA, Cay20292), α-muricholic acid (α-MCA, Cay20291), ursodeoxycholic acid (UDCA, AG-CN2-0411), deoxycholic acid (DCA, Cay20756), lithocholic acid (LCA, Cay20253), chenodeoxycholic acid (CDCA, AG-CN2-0410), glycocholic acid (G-CA, Cay20276), glycoursodeoxycholic acid (G-UDCA, Cay21698), glycochenodeoxycholic acid (G-CDCA, Cay16942), glycodeoxycholic acid (G-DCA, Cay20274), glycolithocholic acid (G-LCA, Cay21723), tauro-ω-muricholic acid (T-ω-MCA, Cay28842), tauro-α-muricholic acid (T-α-MCA, Cay20288), tauro-β-muricholic acid (T-β-MCA, Cay20289), taurocholic acid (T-CA, Cay16215), tauroursodeoxycholic acid (T-UDCA, Cay20277), taurochenodeoxycholic acid (T-CDCA, Cay20275), taurodeoxycholic acid (T-DCA, Cay15935), and taurolithocholic acid (T-LCA, Cay17275) all were obtained from Biomol (Hamburg, Germany), and β-muricholic acid (β-MCA, SML2372), cholic acid (CA, C1129), and 7-keto deoxycholic acid (7-keto DCA, SMB00806) were purchased from Sigma-Aldrich (Taufkirchen, Germany). The four internal standards (IS), namely lithocholic acid-d4 (LCA-d4, Cay20831), taurocholic acid-d4 (T-CA-d4, Cay21891), deoxycholic acid-d4 (DCA-d4, Cay20851), and cholic acid-d4 (CA-d4, Cay20849) all were from Biomol. HPLC grade ultra-pure water (1153332500), methanol (1060352500), acetonitrile (ACN, 1000292500), and formic acid (FA, 5438040100) were from Merck (Darmstadt, Germany). Oasis HLB cartridges (30 mg/1 mL) were purchased from Waters (Milford, MA).

### 2.2 Animals

After approval by the Ethics Committee for animal welfare, Giessen, Germany (V 54-19 c 20 15 h 01 GI 18/17), patient acquisition was performed between November 2021 and June 2022. For study participation, all owners gave their written consent. Suitable dogs comprised adult healthy individuals and pups of all breeds, aged one to ten years and two months to one year, respectively, with a minimum body weight of 3.5 kg. Prior to study application, owners were required to fill in a questionnaire regarding general health (e.g., feeding modalities, husbandry, vaccination, deworming, previously diagnosed diseases, and medication) and specific questions with respect to gastrointestinal health (e.g., fecal consistency, defecation frequency, vomiting, body weight). In the questionnaire, feeding modalities were grouped into five categories: commercial dog food (dry and canned food) only, home-made diet only, raw meat-based diet only, mixed diets, and vegan/vegetarian diet only. Vegan/vegetarian fed dogs were excluded from this study. However, more detailed analytical food analysis or food intake recording have not been performed. The health status was assessed by a board-certified specialist for small animal internal medicine (A-LP) by the combination of an extended routine physical examination and basic laboratory analysis (hematology, biochemistry profile, urinalysis including UP/C). Abnormalities found at clinical examinations, basic laboratory analysis and/or the questionnaire led to preclusion from the study. In addition, systemic antibiotic treatment within the last 6 months prior to this study led to exclusion.

Prior to blood sampling, pups and adults were fasted for at least 8 and 12 h, respectively. Depending on dog size, venipuncture was performed either at the cephalic or saphenic vein. For study purposes, blood was allowed to drop directly into EDTA or no medium containers. Contamination between containers was strictly avoided. Serum samples were allowed to clot for 30 min before centrifugation, while EDTA samples were centrifuged directly. All blood specimens were subject to centrifugation for one min at 100,000 rpm (diameter of centrifuge 86 mm) and supernatants were aliquoted to 0.5 mL samples prior to freezing. Sterile urine samples were obtained by ultrasound guided cystocentesis and immediately aliquoted (0.5 mL) and frozen after acquisition. Freely passed fecal samples were accepted if collected either the day of or, at maximum, the day prior to examinations. Owners stored fecal samples at room temperature until the study appointment. All blood and urine samples were immediately frozen and stored at −80°C until further processing. After receiving the fecal samples, fecal samples were immediately frozen and stored at −80°C in most cases with some fecal samples stored at −20°C.

In total, 40 healthy adult dogs and 10 healthy pups of different breeds were included in the present study. Most of the dogs were mixed breeds (50%) and golden retriever (10%) (Table 1). The adults were of mixed sex and castrate status and pups were only female (Table 2). The adults ranged in body weight from 4.9 to 53.9 kg and averaged 21.4 ± 10.0 kg, whereas the pups ranged in body weight from 5.5 to 28.4 kg and averaged 18.6 ± 7.5 kg. The animal ages ranged from 2 to 6 years for the adults and 5.5 to 9 months for the pups (Table 3 and Supplementary Figure S1).

**Table 1:** Breeds of study population.

	Breed	Number	%
Adult *n* = 40	Mix	20	50
	Golden retriever	4	10
	Australian shepherd	2	5
	Nova scotia duck tolling retriever	2	5
	Vizsla	2	5
	Old German herding dogs	1	2.5
	Bichon frise	1	2.5
	Border collie	1	2.5
	French bulldoge	1	2.5
	Miniature poodle	1	2.5
	Labrador retriever	1	2.5
	Malinois	1	2.5
	Mudi	1	2.5
	Tibetan Terrier	1	2.5
	Toy poodle	1	2.5
Pup *n* = 10	Border collie	1	10
	Dackel	1	10
	German shepherd dog	1	10
	Giant Schnauzer	1	10
	Golden retriever	1	10
	Great dane	1	10
	Irish red setter	1	10
	Labrador retriever	1	10
	Malinois	1	10
	Pug	1	10

**Table 2:** Sex and neuter status of study population.

	Sex	Number	%
Adult	Female entire	11	27.5
	Female neutered	7	17.5
	Male entire	11	27.5
	Male neutered	11	27.5
Pup	Female entire	10	100

**Table 3:** Age and body weight status of study population (mean ± SD).

	Adult			Pup		
Age	3.3	±	1.3 (years)	7.4	±	1.4 (months)
Weight (kg)	21.4	±	10.0	18.6	±	7.5

### 2.3 Sample preparation

**Figure 2:**
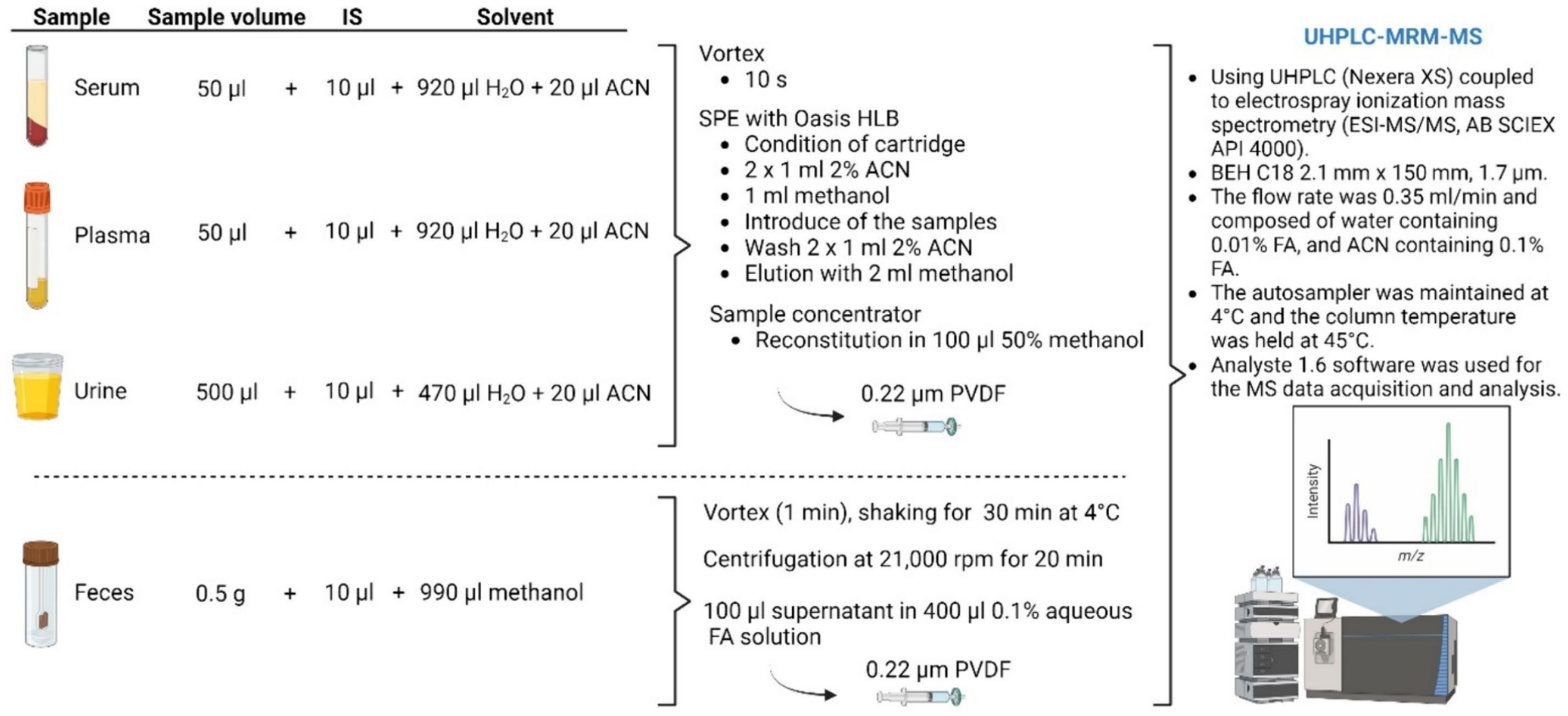
Illustration of the sample preparation method for BA analysis. IS, internal standard; SPE, solid phase extraction; ACN, acetonitrile; FA, formic acid. Figure created with BioRender.

Serum, plasma, urine, or feces samples were stored at −80°C until measurement and thawed at room temperature before processing. The sample preparation process of serum, plasma, and urine (see Figure 2) was performed according to a previously reported method (38). Briefly, 50 μL of either serum or plasma were mixed with 10 μL of the IS solution containing four d4-labeled BA (final concentration: 500 ng/mL each), 920 μL of water, and 20 μL of ACN, finally representing 2% ACN. Similarly, 500 μL of urine were mixed with 10 μL of the IS solution, 470 μL of water, and 20 μL of ACN, finally representing 2% ACN. After mixing by vortex for 10 s, samples were subjected to solid-phase extraction (SPE) using Oasis HLB (30 mg/1 mL) SPE cartridges. The SPE cartridges were placed on a chromabond SPE vacuum manifold from Macherey-Nagel (Düren, Germany). Before use, the cartridges were conditioned with 2 × 1 mL 2% aqueous ACN followed by 1 mL methanol. Then, samples were introduced into the cartridges and the cartridges were washed with 2 × 1 mL of 2% aqueous ACN. Finally, the analytes were eluted with 2 mL of methanol and dried using a sample concentrator (Techne, Cambridge, United Kingdom) at room temperature with a gentle stream of nitrogen. The residues were reconstituted in 100 μL of 50% methanol. Fecal samples were prepared following a method previously reported by Shafaei et al. (39). Briefly, 0.5 g of wet fecal samples were weighed into tubes and diluted with 1 mL ice-cold methanol containing the IS (final concentration: 500 ng/mL each). The samples were capped and shortly vortex mixed. The mixtures were shaken for 30 min at 4°C, briefly vortexed mixed again, and centrifuged at 21,000 rpm for 20 min. The supernatant solution (100 μL) was diluted with 400 μL of 0.1% aqueous formic acid (FA) solution. For all samples, the extract was then filtered through 0.22 μm polyvinylidene difluoride (PVDF) syringe filters and transferred to 2 mL vials with 0.1 mL clear glass micro insert for analysis. For each sample, 10 μL were injected for UHPLC/MRM-MS.

### 2.4 Analysis using UHPLC-MRM/MS

Dog BA profiling in serum, plasma, urine, and feces was performed by reverse-phase UHPLC multiple-reaction monitoring-mass spectrometry (UHPLC-MRM/MS) with negative ion detection using Nexera XS inert UHPLC (Shimadzu, Kyoto, Japan) coupled with API 4000 mass spectrometer from Applied Biosystems (AB Sciex, Framingham, MA, United States) according to a published procedure (38). Chromatographic separation was performed using a BEH C18 analytical column (2.1 mm × 150 mm, 1.7 μm particle size) coupled to a VanGuard BEH C18 pre-column (2.1 mm × 5 mm, 1.7 μm particle size) both from Waters (Milford, MA, United States). The flow rate was 0.35 mL/min and composed of solvent A (water containing 0.01% FA), and solvent B (ACN containing 0.01% FA). The chromatographic gradient is shown in Table 4. The autosampler was kept at 4°C, and the column temperature was maintained at 45°C. A representative chromatogram is presented in Supplementary Figure S2, and the MRM transitions and MS parameters are listed in Supplementary Table S1. MS data acquisition and analysis were performed using the Analyste 1.6 software (AB Sciex). From all urine samples, the creatinine concentrations were additionally determined by Synlab (Augsburg, Germany) for normalization of the urinary BA concentrations.

**Table 4:** Chromatographic gradient parameters.

Number	Time	Flow	A (H_2_O)	B (ACN)
1	0.00 min	0.35 mL/min	75%	25%
2	12.00 min	0.35 mL/min	60%	40%
3	26.00 min	0.35 mL/min	25%	75%
4	26.01 min	0.35 mL/min	0%	100%
5	28.00 min	0.35 mL/min	0%	100%
6	28.01 min	0.35 mL/min	75%	25%
7	32.00 min	0.35 mL/min	75%	25%

### 2.5 Statistics

Graphs and calculations were generated using GraphPad Prism software 6.07 (GraphPad Software, La Jolla, CA, United States). All variables were tested for normal distribution using the Kolmogorov–Smirnov test. The Student’s *t*-test was used to compare the means of continuous variables and normal-distributed data. When data were skewed, medians were compared with the Mann–Whitney U test. All data were expressed as mean ± standard deviation (SD). A level of *p* < 0.05 was considered statistically significant.

## 3 Results

### 3.1 Bile acid concentrations and profiles in serum and plasma

The composition of BA in the blood, urine, and feces of forty adult dogs and ten pups was analyzed using LC-MS/MS. The results are summarized in Table 5 and Supplementary Figure S3 for the adult dogs, and in Table 6 and Supplementary Figure S4 for the pups. Additionally, this study investigated differences in BA analytics of serum and plasma samples from the same individual dogs. In general, the BA serum profile closely resembled the plasma profile in both adult and pup groups. A statistically significant difference was only observed for LCA between serum (0.57 ± 0.07 μmol/L) and plasma (0.70 ± 0.09 μmol/L) in the pup group. In adult dogs, the total BA concentrations in serum and plasma were 29.62 μmol/L and 26.50 μmol/L, respectively (Table 5), both displaying large interindividual variations. In comparison, the concentration of total BA in pups was notably lower, with values of 10.85 μmol/L in serum and 10.80 μmol/L in plasma (Table 6).

**Table 5:** Serum, plasma, urine, and feces BA concentrations in 40 adult dogs (mean ± SD).

		Adult																
BA		Serum (μmol/L)				Plasma (μmol/L)					Urine (μmol/mg Cr)				Feces (μmol/g)			
		Mean		SD	*n*	Mean		SD	*n*	p*	Mean		SD	*n*	Mean		SD	*n*
Unconjugated	ω-MCA	0.417	±	0.319	7	0.499	±	0.410	3		0.078	±	0.066	10	n.d.			0
	α-MCA	0.423	±	0.452	9	0.309	±		1		0.151	±	0.077	9	3.528	±	5.329	31
	β-MCA	0.229	±	0.331	18	0.181	±	0.082	3		0.271	±	0.370	32	2.690	±	4.363	32
	CA	18.269	±	23.510	8	13.597	±	18.945	9		5.344	±	7.734	26	58.088	±	139.699	32
	UDCA	0.319	±	0.396	34	0.257	±	0.231	38		0.094	±	0.139	23	18.130	±	36.487	32
	DCA	1.930	±	3.058	40	1.976	±	2.664	40	0.942	0.164	±	0.159	40	1412.999	±	956.745	32
	LCA	0.565	±	0.558	40	0.661	±	0.190	40	0.307	0.260	±	0.607	40	96.464	±	198.567	32
	7-keto DCA	0.221	±	0.386	40	0.215	±	0.072	17		1.321	±	2.540	40	55.253	±	136.818	32
	CDCA	0.473	±	0.812	40	0.442	±	0.742	40	0.858	0.070	±	0.162	40	68.134	±	106.416	32
G-conjugated	G-CA	0.448	±	0.240	5	0.300	±	0.014	2		0.119	±	0.086	13	1.292	±	3.526	28
	G-UDCA	0.756	±	0.104	2	n.d.			0		0.019	±	0.014	12	0.036	±	0.031	31
	G-CDCA	0.344	±	0.311	8	0.114	±	0.094	7		0.175	±	0.202	11	0.198	±	0.366	27
	G-DCA	0.315	±	0.312	10	n.d.			0		0.198	±	0.193	5	0.903	±	1.517	32
	G-LCA	0.534	±	0.605	9	0.120	±	0.055	39		0.224	±	0.411	12	0.309	±	0.341	15
T-conjugated	T-ω-MCA	n.d.			0	n.d.			0		n.d.			0	n.d.			0
	T-α-MCA	n.d.			0	n.d.			0		n.d.			0	1.875	±	2.308	3
	T-β-MCA	n.d.			0	n.d.			0		n.d.			0	n.d.			0
	T-CA	10.752	±	9.655	40	9.804	±	8.946	40	0.650	2.189	±	2.163	40	3.121	±	6.811	32
	T-UDCA	0.463	±	0.482	24	0.393	±	0.442	23		0.076	±	0.164	31	0.109	±	0.101	19
	T-CDCA	4.825	±	8.703	32	4.329	±	8.299	32		0.099	±	0.097	26	2.311	±	5.753	25
	T-DCA	6.376	±	11.001	40	6.061	±	11.353	40	0.900	0.059	±	0.085	39	11.267	±	27.108	32
	T-LCA	0.607	±	2.311	40	0.264	±	0.405	40	0.358	0.185	±	0.321	27	0.811	±	0.883	26
Total	Total	29.618	±	32.799	40	26.503	±	29.650	40		8.292	±	10.497	40	1734.649	±	1142.630	32
	Unconj	7.385	±	17.228	40	6.533	±	13.648	40		5.613	±	9.098	40	1715.176	±	1117.867	32
	G-conj.	1.112	±	1.440	13	0.156	±	0.112	39		0.369	±	0.660	20	2.380	±	5.070	32
	T-conj.	21.872	±	24.410	40	19.818	±	24.346	40		2.494	±	2.295	40	17.093	±	39.582	32
	Primary	19.061	±	22.056	40	16.825	±	19.554	40		6.135	±	8.357	40	138.730	±	261.230	32
	Secondary	10.557	±	13.099	40	9.679	±	12.561	40		2.157	±	2.857	40	1595.919	±	1054.668	32

*The *p* values were only calculated for comparisons of groups with 40 samples for the respective individual BA between serum and plasma. The bile acids α-MCA, β-MCA, CA, CDCA, and their conjugates with taurine (T) or glycine (G) were regarded as primary BA, while all others were regarded as secondary BA. n.d., not detectable. Some individual BA could not be detected in all dogs. Accordingly, mean values for the individual BA refer to the indicated amount of data (*n*). For the total BA concentrations and the respective subgroups (unconjugated, conjugated, primary, secondary), mean values were calculated from the total BA concentrations of each individual dog (*n* = 40 for serum, plasma, and urine samples; *n* = 32 for feces samples). Cr, creatinine.

**Table 6:** Serum, plasma, urine, and feces BA concentrations in 10 pups (mean ± SD).

		Pup																
BA		Serum (μmol/L)				Plasma (μmol/L)					Urine (μmol/ mg Cr)				Feces (μmol/g)			
		Mean	SD		*n*	Mean	SD		*n*	*p*	Mean		SD	*n*	Mean	SD		*n*
Unconjugated	ω-MCA	n.d.	0	n.d.			0		0.201	±		1	n.d.	0
	α-MCA	n.d.	0	n.d.			0		0.322	±		1	2.376	±	2.35	10
	β-MCA	0.061	±	0.011	3	n.d.			0		0.156	±	0.144	6	1.769	±	0.86	10
	CA	3.224	±	1.844	2	2.210	±	1.725	2		1.466	±	0.812	6	34.537	±	55.08	10
	UDCA	0.215	±	0.038	10	0.245	±	0.057	10	0.175	0.174	±	0.142	3	14.341	±	6.27	10
	DCA	1.652	±	1.406	10	1.622	±	1.319	10	0.9603	0.617	±	1.134	9	1142.708	±	870.80	10
	LCA	0.571	±	0.065	10	0.695	±	0.090	10	0.0024	0.231	±	0.289	9	116.020	±	135.91	10
	7-keto DCA	0.168	±	0.134	3	0.205	±	0.026	2		0.702	±	0.675	9	30.436	±	42.99	10
	CDCA	0.257	±	0.094	10	0.307	±	0.116	10	0.3074	0.065	±	0.088	9	99.088	±	179.60	10
G-conjugated	G-CA	n.d.			0	n.d.			0		0.140	±		1	1.441	±	2.51	6
	G-UDCA	n.d.			0	n.d.			0		0.065	±		1	0.026	±	0.02	6
	G-CDCA	0.096	±	0.067	4	n.d.			0		0.211	±		1	0.267	±	0.33	6
	G-DCA	n.d.			0	n.d.			0		0.194	±		1	0.563	±	0.67	10
	G-LCA	0.102	±	0.008	7	0.128	±	0.028	10		0.163	±		1	0.171	±	0.07	4
T-conjugated	T-ω-MCA	n.d.			0	n.d.			0		n.d.			0	n.d.			0
	T-α-MCA	n.d.			0	n.d.			0		n.d.			0	3.076	±		1
	T-β-MCA	n.d.			0	n.d.			0		n.d.			0	n.d.			0
	T-CA	3.981	±	3.527	10	3.245	±	2.892	10	0.6161	1.252	±	1.045	9	48.043	±	142.48	10
	T-UDCA	0.139	±		1	n.d.			0		0.042	±	0.035	6	0.099	±	0.10	6
	T-CDCA	1.638	±	2.281	6	1.793	±	2.578	7		0.111	±	0.102	6	5.693	±	14.07	9
	T-DCA	2.257	±	3.728	10	2.691	±	4.650	10	0.8204	0.050	±	0.060	9	14.440	±	38.82	10
	T-LCA	0.093	±	0.061	10	0.130	±	0.062	10	0.1958	0.089	±	0.233	9	1.613	±	3.21	7
Total	Total	10.846	±	10.229	10	10.801	±	10.152	10		4.393	±	4.283	9	1512.050	±	885.67	10
	Unconj	3.408	±	2.397	10	3.352	±	1.701	10		2.813	±	3.190	9	1441.275	±	882.10	10
	G-conj.	0.137	±	0.071	8	0.128	±	0.028	10		0.387	±	0.349	2	1.672	±	2.73	10
	T-conj.	7.327	±	8.535	10	7.321	±	9.093	10		1.493	±	1.157	9	69.103	±	198.38	10
	Primary	5.923	±	5.598	10	5.249	±	4.654	10		2.548	±	2.101	9	192.270	±	270.08	10
	Secondary	4.923	±	5.085	10	5.552	±	5.953	10		1.845	±	2.263	9	1319.780	±	836.31	10

*The *p* values were only calculated for comparisons of groups with 10 samples for the respective individual BA between serum and plasma. The bile acids α-MCA, β-MCA, CA, CDCA, and their conjugates with taurine (T) or glycine (G) were regarded as primary BA, while all others were regarded as secondary BA. n.d., not detectable. Some individual BA could not be detected in all dogs. Accordingly, mean values for the individual BA refer to the indicated amount of data (*n*). For the total BA concentrations and the respective subgroups (unconjugated, conjugated, primary, secondary), mean values were calculated from the total BA concentrations of each individual dog (*n* = 10). Cr, creatinine.

**Figure 3:**
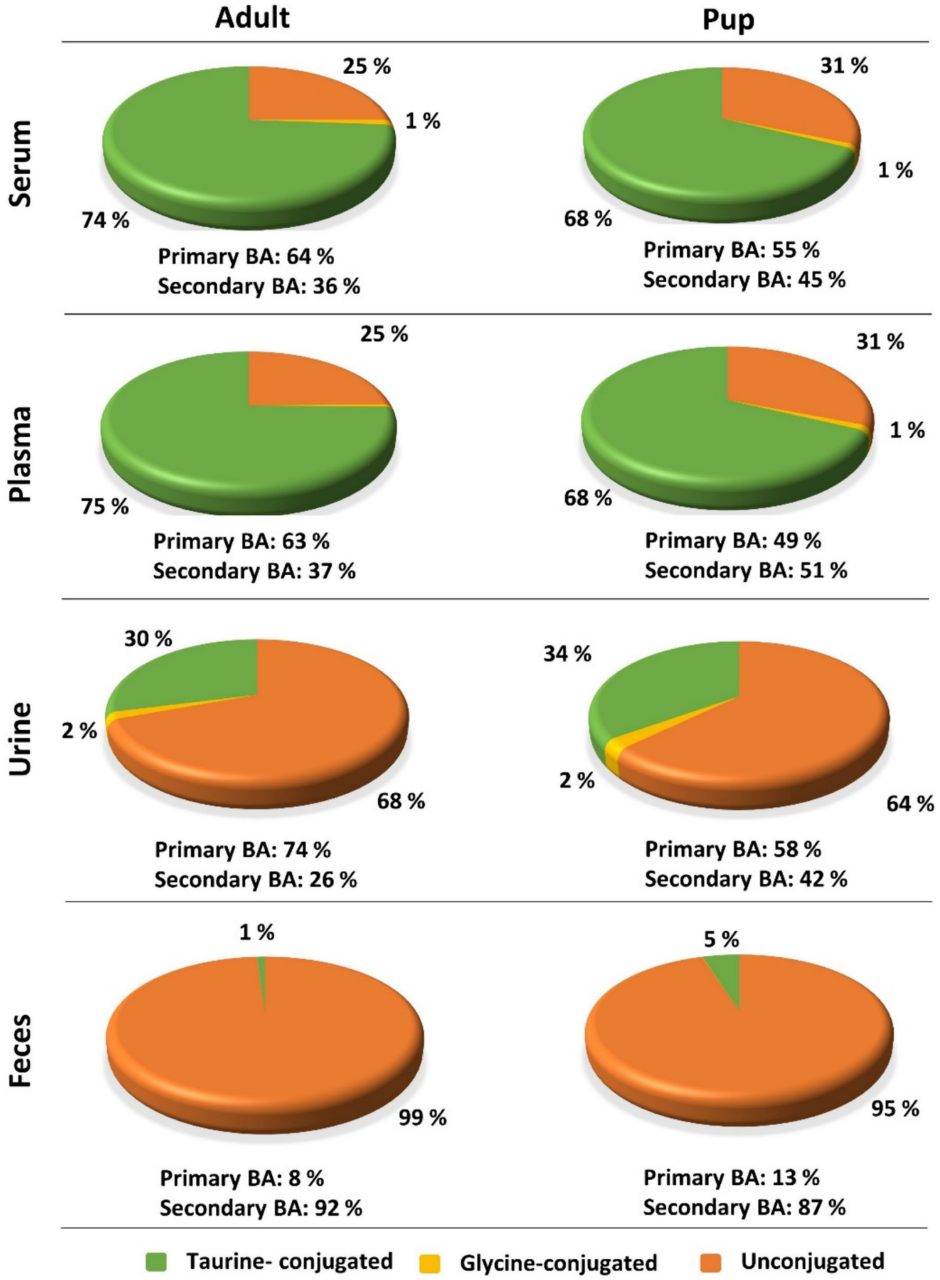
Contributions of taurine-conjugated (green), glycine-conjugated (yellow), and unconjugated (orange) BA to the overall BA composition of serum, plasma, urine, and feces in adult dogs and pups. Percentage values were calculated based on the total BA concentrations listed in Table 5 (adult) and Table 6 (pup).

**Figure 4:**
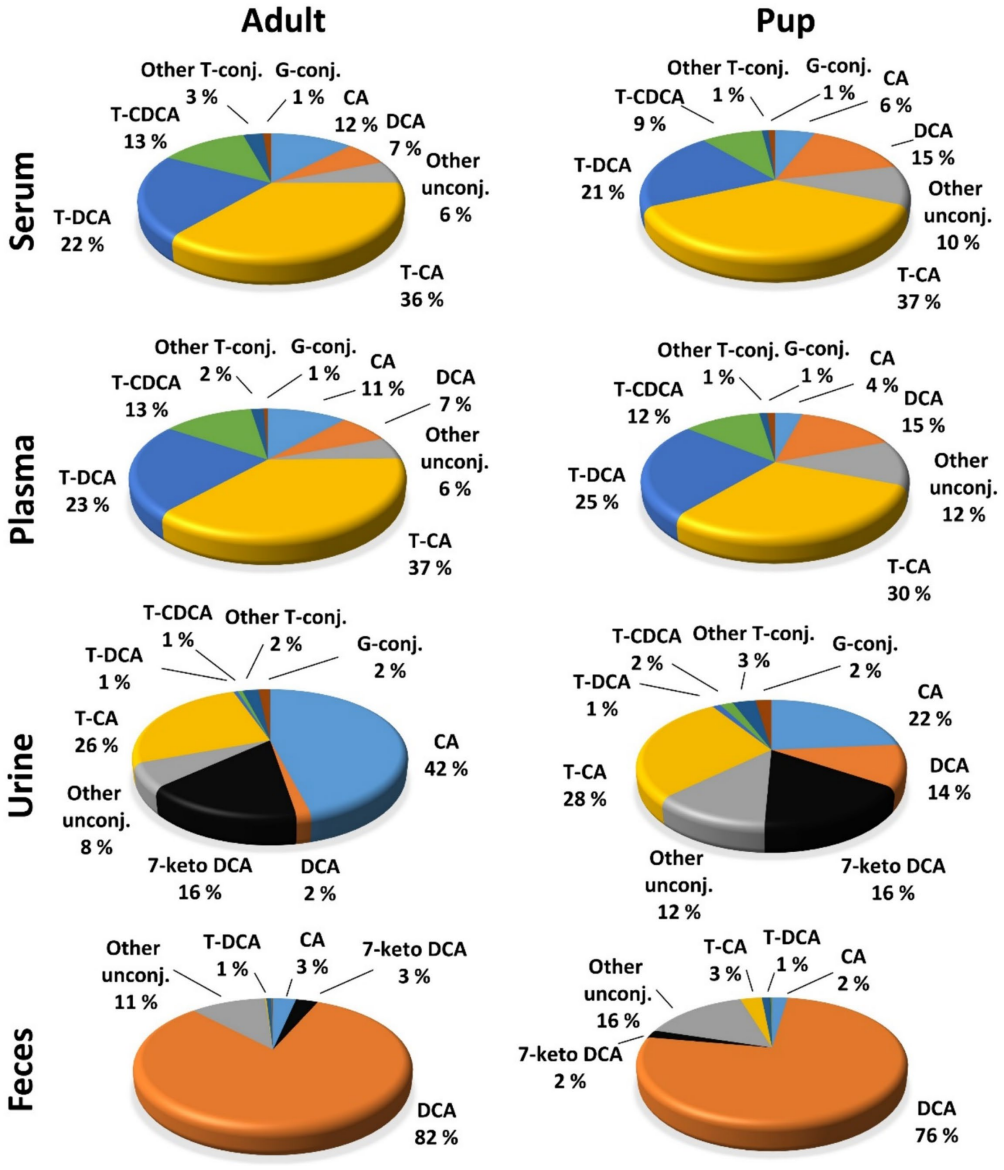
Contributions of individual BA to the overall BA composition of serum, plasma, urine, and feces BA in adult dogs and pups. Percentage values were calculated for each individual BA as means of the total sample number (*n* = 40 for serum, plasma, and urine samples; *n* = 32 for feces samples in adults; *n* = 10 for pup) and were related to the total BA concentration.

Regarding the BA composition in serum, 74% of the BA in adult dogs were taurine conjugates, 25% were unconjugated, and 1% were glycine conjugates (Figure 3). The most abundant BA in adult serum were T-CA (36%), T-DCA (22%), T-CDCA (13%), CA (12%), and DCA (7%) (Figure 4). In the pup group, 68% of BA in serum were taurine conjugates, 31% were unconjugated, and 1% were glycine conjugates (Figure 3). The predominant BA in pup serum were basically the same as in the serum of adult dogs, namely T-CA (37%), T-DCA (21%), T-CDCA (9%), CA (6%), and DCA (15%) (Figure 4).

In adult dogs, the concentrations of primary BA in serum (19.06 μmol/L) or plasma (16.83 μmol/L) were nearly double those of secondary BA (10.56 μmol/L and 9.68 μmol/L, respectively) (Table 5). In contrast, in the pups, levels of primary and secondary BA were similar in both serum and plasma (Table 6). Of note, CA was the predominant unconjugated BA in the serum and plasma of some dogs (*n* = 8 and *n* = 9, respectively), while CA was undetectable in the serum and plasma samples of most of the other dogs. A similar pattern was observed for the pups, where CA was detectable only in the serum and plasma samples of two out of ten pups. However, taurine conjugated cholic acid (T-CA) was detected as the predominant conjugated BA in all serum and plasma samples of all adult dogs and pups. In contrast, the other primary BA, CDCA, was detected in the serum and plasma samples from all dogs (*n* = 40 adult and *n* = 10 pup). In this case, the concentration of T-CDCA far exceeded the concentration of unconjugated CDCA in all samples (e.g., 4.83 vs. 0.47 μmol/L in serum of adult dogs and 1.64 vs. 0.26 μmol/L in serum of pups).

Within the group of secondary BA, DCA dominated in both unconjugated and taurine-conjugated form. Even more, the serum DCA and T-DCA concentrations were the second highest concentrations in their respective conjugation group, with 1.93 μmol/L DCA and 6.38 μmol/L T-DCA in the adults and 1.65 μmol/L DCA and 2.26 μmol/L T-DCA in the pups. The muricholic acids (ω-MCA, α-MCA, and β-MCA) were detected in a limited number of adult serum and plasma samples. Notably, only three serum samples from pups showed the presence of β-MCA, while T-ω-MCA, T-α-MCA, and T-β-MCA were not detected in serum or plasma samples of either adult dogs or pups.

### 3.2 Bile acid concentrations and profiles in urine

**Figure 5:**
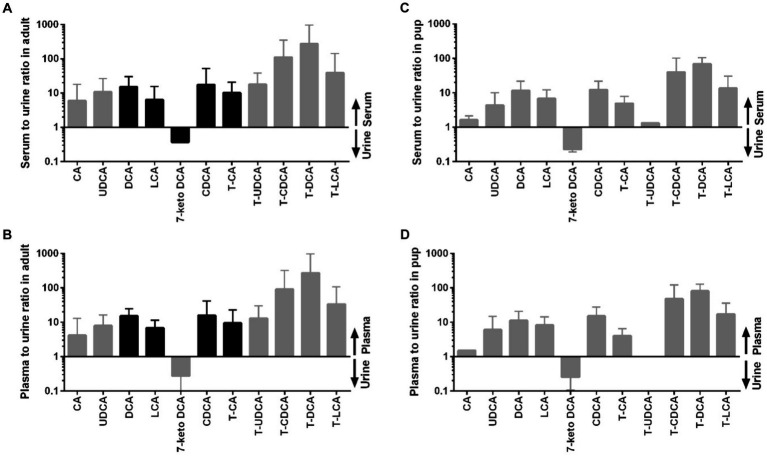
Distribution of individual BA between serum or plasma and urine in adult dogs and in pups. **(A)** Serum to urine ratio in adult dogs, **(B)** plasma to urine ratio in adult dogs, **(C)** serum to urine ratio in pups, and **(D)** plasma to urine ratio in pups. Black bars represent datasets of *n* = 40 in the adult dogs, whereas datasets <40 in the adults or <10 in the pups are indicated by gray bars.

Whereas the conjugated BA clearly dominated in the serum and plasma samples, unconjugated BA were much more prevalent in urine. The urinary total BA concentrations in adult dogs were 8.29 μmol/mg Cr (Table 5) and were notably lower in the pups with a mean value of 4.39 μmol/mg Cr (Table 6). Unfortunately, only nine out of the ten urine samples from the pups could be analyzed, and one sample did not provide any data for unknown reason. Regarding the BA composition in the urine of adult dogs, 68% of the BA were unconjugated, 30% were taurine conjugates, and less than 2% were glycine conjugates (Figure 3). These values were quite similar in the pup group. The most abundant individual BA in urine samples from adult dogs were CA (42%), T-CA (26%), and the secondary BA 7-keto DCA (16%) (Table 5 and Figure 4). The most abundant primary BA detected in urine of the pups were T-CA (28%) and CA (22%). In addition, 7-keto DCA and DCA were present with 16 and 14% of the total BA concentration, respectively (Table 6 and Figure 4). The most significant difference in the BA profile between blood and urine was the dominant occurrence of 7-keto-DCA in the urine, whereas this BA showed very low concentrations in the blood. Accordingly, when looking at the serum-to-urine or plasma-to-urine ratios of the individual BA (Figure 5), 7-keto DCA was the only BA that consistently showed higher urine than serum or plasma concentrations. A second important observation was that taurine-conjugated BA overall showed the highest serum-to-urine or plasma-to-urine ratios, clearly indicating that taurine-conjugated BA are sparsely excreted from the blood into the urine.

### 3.3 Bile acid concentrations and profiles in feces

For the present study, only 32 feces samples were available from the 40 adult study dogs. In the adult dogs, the total BA concentrations in the feces were at 1734.64 μmol/g, predominantly composed of 99% unconjugated BA. The pups exhibited total BA concentrations of 1512.05 μmol/g in feces, with 95% representing unconjugated BA (Figure 3). In both study groups, DCA was the predominant component in fecal samples of both adult dogs (82%) and pups (76%), followed by LCA, CDCA, CA, and 7-keto DCA (Figure 4 and Tables 5, 6). Notably, β-MCA could be detected and quantified in all analyzed feces samples, in contrast to serum, plasma, and urine.

**Figure 6:**
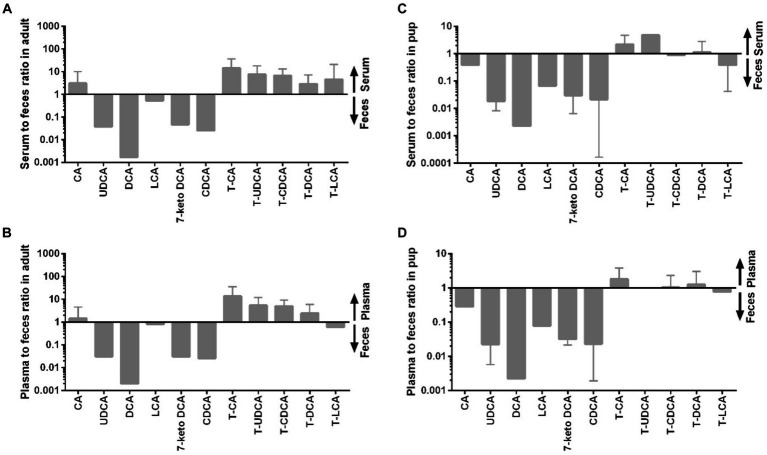
Distribution of individual BA between serum or plasma and feces in adult dogs and in pups. **(A)** Serum to feces ratio in adult dogs, **(B)** plasma to feces ratio in adult dogs, **(C)** serum to feces ratio in pups, and **(D)** plasma to feces ratio in pups.

Figure 6 provides additional information on the distribution of individual BAs between blood and feces. Of note, concentrations of the unconjugated mostly secondary BA clearly dominated in the feces samples (DCA, 7-keto DCA, LCA, UDCA), whereas the taurine-conjugated primary BA showed the highest blood-to-feces ratios.

## 4 Discussion

In the context of several previous BA profiling studies in dogs, the present study particularly focused on the following three aspects: (I) comparison of BA concentrations and profiles between randomly recruited adult dogs and pups from the patient collective of an university veterinary clinic, consciously not representing uniform study groups; (II) in parallel determination of BA concentrations and profiles in blood, urine and feces samples for each individual dog; and (III) direct comparison of the BA measurements from serum and plasma samples from each individual dog. All these points are relevant considering the potential diagnostic use of selected BA as biomarkers for liver diseases (21, 23, 24, 32, 40). For this, it is important to get an impression about the variation of BA concentrations between dogs of different breeds and different age groups. Moreover, determining which matrix (plasma, serum, urine, feces) is the most suitable for diagnostic BA measurements is essential (29). While it is impossible to get bile samples for biomarker studies and blood samples still require invasive venipuncture, the analysis of urine or feces is an attractive non-invasive alternative (31, 37, 41). Finally, there is no general consent in previous BA profiling studies regarding whether plasma or serum is the most appropriate matrix to analyze blood levels of BA (22, 29–32, 37, 41). Considering all these points, the present study contributes several important information to the field.

(I) There is a great interindividual variation for the concentrations of single BA, mostly exemplified by the fact that CA is by far the most prominent BA in blood and urine samples of some of the dogs (adults and pups), while in others CA was below the limit of detection. This indicates that for heterogeneous patient collectives, the BA with the highest concentration may not necessarily be the best choice for diagnostic BA measurements. This finding is in general agreement with previous studies (31, 41, 42). The interindividual BA variations identified in the present study could be of genetic origin and so might just reflect the different genetics of different dog breeds. In addition, the non-standardized feeding of the study population and the presumed individual microbiota composition (see below) might account for these differences. However, detailed diet and microbiome compositions have not been analyzed in the present study, so that correlation studies could not be performed.

(II) While the BA profiles were quite similar between the adult dogs and the pups for all analyzed matrices, there were significant age-related differences in the absolute amount of single BA, limiting the use of diagnostic BA determination with the same reference values for all age groups. In the present study, total BA concentration in plasma and serum were higher in adult dogs than in pups (Tables 5, 6). In addition, the adult dogs had a higher proportion of taurine-conjugated vs. unconjugated BA and a dominance for primary vs. secondary BA (Figure 3). Similarly, a study in rats showed significant increase of T-CA with age (43). However, a previous study using UHPLC-Q-TOFMS-based metabolomics to examine plasma samples from 15 young and 15 adult beagle dogs showed higher plasma levels for the young beagle dogs, at least for T-CA, G-CA, and T-CDCA (41). This difference might be explained by the study collective (only beagle dogs vs. diverse dog breeds in the present study) or by differences in the absolute age of the groups of young and adult dogs. Based on this, the effect of age on the absolute BA concentrations in dogs needs further investigation.

(III) It must be carefully considered which sampling is most appropriate for diagnostic BA measurements. While bile, as the matrix, best reflects the composition of the entire BA pool, bile sampling requires general anesthesia or deep sedation and, thus, cannot be achieved during normal clinical examinations. The concentrations of blood BA largely depend on active BA transport processes in the liver, which are more relevant for hydrophilic conjugated BA than for unconjugated BA (13, 44). Therefore, blood BA concentrations do not reflect the actual composition of the BA pool. On the other hand, blood BA levels typically increase under different liver disease conditions due to impaired uptake of BA into the liver and hampered canalicular efflux into bile (45). Therefore, blood is a valuable matrix for liver diagnostics. Urine BA concentrations and profiles do not well reflect the actual BA pool, as most conjugated BA are actively reabsorbed in the proximal tubules and only a small fraction of approximately 5% of the renally filtered BA enters the urine (23, 29, 30, 46, 47). However, in cases of experimental and clinical cholestatic liver diseases, urinary excretion becomes the primary route for BA elimination and, therefore, urinary BA are attractive diagnostic markers for liver diseases (48). Finally, feces BA do not primarily reflect the physiological BA pool but are more dependent on the metabolic activity of the microbiota, which typically exhibits large interindividual variability (10, 49–51). Of note, as many secondary BAs that result from the microbial degradation of primary BAs are reabsorbed and thus also appear in the blood, the blood compartment reflects the hepatic capacity of BA synthesis, conjugation, and transport, and in addition the metabolic activity of the microbiome (9, 36, 52). In this context, the determination of DCA and its taurine-conjugated form (T-DCA), both of which could be robustly detected in all dogs in blood and feces, are interesting candidates for diagnostic BA measurements. They reflect the BA synthesis capacity of the liver (synthesis of CA), its microbial conversion to DCA, and finally, the re-conjugation capacity of the liver to T-DCA. However, taurine-conjugation can also be affected by the diet as diets low in sulfur amino acids might influence the taurine pool in some dogs, especially in large dog breeds and hence could secondarily reduce taurine-conjugation of BA (53). Animal products, especially meat and seafood, are the major source of taurine. The dogs in our study were not fed a standardized study diet, and so the exact dietary taurine contents were not known. In addition, blood taurine concentrations were not measured. All dogs were fed a meat-based diet and most of the dogs received either commercial dog food only or a diet comprising meat and commercial dog food and, thus, taurine supply should be sufficient. However, taurine deficiency can occur in individual dogs even when fed a commercial meat-based diet (54). Therefore, a nutritional effect on the taurine-conjugation of BA cannot completely be excluded.

Intestinal bacteria can produce keto-bile acids, such as 7-keto DCA, through a sequence of enzymatic deconjugation, dehydroxylation, and oxidoreduction reactions (52). Thereby, 7-keto DCA originates from CA/T-CA (55). Zhang et al. (56) demonstrated an increase in serum CA and DCA levels in Oatp1a4 bile acid transporter knockout mice. In addition, they showed higher serum 7-keto DCA levels after bile duct ligation in these knockout mice. In another study, 7-keto DCA increased in the urine of rats with acrylamide-induced liver toxicity, indicating the potential role of 7-keto DCA as a urinary biomarker for assessing liver function and toxicity (57, 58). Of note, 7-keto DCA was one of the most prominent BA in the urine in the present study (Figure 4) and could robustly be detected in all urine samples (Table 5). Moreover, 7-keto DCA was the only BA with higher occurrence in the urine as in the plasma/serum of the dogs (Figure 5), underlining its potential role as urinary biomarker. However, Choucair et al. (59) observed a statistically significant difference in 7-keto DCA levels between male and female mice in both plasma and feces, which may limit its diagnostic potential at least in mice.

(IV) A variety of matrices, including serum, plasma, bile, urine, tissue homogenate, and feces, are commonly used as biological samples for the detection of BA. Whether serum and plasma can be considered interchangeable for the quantification of BA was investigated in a recent study by Sangaraju et al. (29). This study revealed no differences between human serum and plasma for the quantification of different BA. Similarly, the present study clearly demonstrates no statistically relevant differences in the BA concentrations measured from serum or plasma from adult dogs and pups. There was only one exception in the case of LCA that showed significantly higher values when plasma was used for LC-MS/MS BA measurements compared to serum (Table 6). An explanation for this effect could be that LCA binds to some extend to clotting factors and so more LCA is separated during serum preparation as during plasma preparation. However, the difference between plasma (0.70 ± 0.09 μmol/L) and serum (0.57 ± 0.07 μmol/L) LCA concentrations was quite low. Nevertheless, based on this finding LCA and its conjugates should not be considered for diagnostic BA quantification from dog plasma or serum samples.

The BA profiles in our study are consistent with previous studies. For example, Washizu et al. (42) measured the BA composition in various species, including dogs, and found that T-CA, T-DCA, and T-CDCA were predominant in dogs. In our study, adult dogs exhibited 37% T-CA, 22% T-DCA, and 13% T-CDCA (Figure 4). However, Washizu et al. (42) reported much lower levels of CA and DCA compared to the present study. Sangaraju et al. (29) also examined BA concentrations and found high percentage of taurine-conjugated BA in dogs, which is like the findings of the present study. However, the ratio of primary to secondary BA in urine varies across studies (29, 30).

Profiling of feces samples from 32 adult dogs and 10 pups allowed the detection of 22 different BA species (Tables 5, 6). Only few previous studies analyzed BA concentration in feces of dogs by MS (3, 34, 51, 60). In accordance with these previous studies, we found >95% of unconjugated BA, predominantly DCA and LCA. Despite the low amount (0.5 g) of feces sample used for analysis in the present study, our protocol using methanol extraction followed by UHPLC/MRM-MS detection allowed detect also of minor BA species such as CA, CDCA and 7-keto DCA and numerous conjugated bile acids (T-CA, T-DCA, T-CDCA, T- UDCA and T-LCA) in the feces (Figure 4 and Tables 5, 6). The considerable interindividual variability in fecal BA concentrations most likely is a result of differences in the microbiome composition and may result from different feeding of the dogs as shown before (34, 36).

It is important to acknowledge the limitations of the present study. Firstly, the number of pups (*n* = 10) included in this study was much lower than that of the adult dogs (*n* = 40), however reflecting the patient collective of a university veterinary clinic. This patient collective was by intention not uniform as in most other studies that mostly used groups of beagle dogs. Consequently, the interindividual variability was relatively high. Assessment of general health was based on the owners’ responses to a questionnaire, clinical examination, and basic laboratory tests including hematology, biochemistry, and urinalysis. However, abdominal ultrasound was not performed. Therefore, possible morphological abnormalities of internal organs may have been missed. Finally, diet that has a large effect on the microbiome and, therefore, on the formation of secondary BA was not standardized. However, as meat-free diets can change the BA pool and BA profiles, feeding of vegetarian/vegan diets led to exclusion from the study. Finally, all dogs from the pup group were female. Consequently, no gender-specific sub-analysis could be performed.

In conclusion, this study provides novel insights into the BA concentrations and profiles in adult dogs and pups, highlighting age-related variations in BA profiles and potential diagnostic implications for canine hepatic and gastrointestinal health. These findings contribute to the expanding field of BA research and may have clinical relevance in veterinary medicine.

## Data availability statement

The LC-MS/MS raw data is deposited in an in-house database operated by the Justus Liebig University Giessen. In order to get access to the data, please contact the corresponding author.

## Ethics statement

The animal studies were approved by Ethics Committee for animal welfare, Giessen, Germany (V 54-19 c 20 15 h 01 GI 18/17). The studies were conducted in accordance with the local legislation and institutional requirements. Written informed consent was obtained from the owners for the participation of their animals in this study.

## Author contributions

EK: Writing – review & editing, Writing – original draft, Visualization, Validation, Methodology, Investigation, Formal analysis, Data curation. A-LP: Writing – review & editing, Writing – original draft, Supervision, Investigation, Formal analysis, Data curation, Conceptualization. AM: Writing – review & editing, Supervision, Resources, Project administration, Formal analysis, Conceptualization. JG: Writing – review & editing, Writing – original draft, Visualization, Supervision, Resources, Project administration, Funding acquisition, Formal analysis, Conceptualization.
