# Supplementary material for: Quantitative bile acid profiling in healthy adult dogs and pups from serum, plasma, urine, and feces using LC-MS/MS
Source: Front Vet Sci. 2024 Jun 14;11:1380920. doi: 10.3389/fvets.2024.1380920 (PMC11211631; doi:10.3389/fvets.2024.1380920)
Supplement: Supplementary file 1 [file Data_Sheet_1.docx]

**SUPPLEMENTARY INFORMATION**

**Quantitative Bile Acid Profiling in Patient Collectives of Adult Dogs and Pups from Serum, Plasma, Urine and Feces using LC-MS/MS**

*Emre Karakus, Anna-Lena Proksch, Andreas Moritz, Joachim Geyer*

**Supplementary Table S1.** MRM transitions and MS parameters for the UHPLC-MRM/MS analysis of BA in the dog.

| **Bile acid** | **ESI** | **Q1 Mass (m/z)** | **Q3 Mass (m/z)** | **DP (*v*)** | **CE (*v*)** | **IS** | **RT (min)** |
| --- | --- | --- | --- | --- | --- | --- | --- |
| ω-MCA | negative | 407.2 | 407.2 | -125 | -48 | CA-d4 | 12.3 |
| α-MCA | negative | 407.3 | 407.3 | -85 | -18 | CA-d4 | 12.9 |
| β-MCA | negative | 407.3 | 407.3 | -155 | -18 | CA-d4 | 13.5 |
| CA | negative | 407.7 | 407.7 | -125 | -18 | CA-d4 | 16.9 |
| UDCA | negative | 391.3 | 391.3 | -135 | -18 | CA-d4 | 17.1 |
| DCA | negative | 391.2 | 391.2 | -125 | -18 | DCA-d4 | 21.6 |
| LCA | negative | 375.1 | 375.1 | -145 | -18 | LCA-d4 | 26.1 |
| 7-keto DCA | negative | 404.9 | 404.9 | -115 | -18 | CA-d4 | 13.5 |
| CDCA | negative | 391.3 | 391.3 | -110 | -18 | DCA-d4 | 21.2 |
| G-CA | negative | 464.3 | 74.0 | -150 | -68 | CA-d4 | 12.8 |
| G-UDCA | negative | 448.4 | 74.1 | -125 | -70 | CA-d4 | 12.4 |
| G-CDCA | negative | 448.3 | 73.7 | -125 | -70 | CA-d4 | 17.4 |
| G-DCA | negative | 448.5 | 74.0 | -105 | -68 | CA-d4 | 18.1 |
| G-LCA | negative | 432.4 | 74.1 | -70 | -68 | DCA-d4 | 22.2 |
| T-ω-MCA | negative | 514.4 | 79.9 | -125 | -128 | T-CA-d4 | 5.7 |
| T-α-MCA | negative | 514.6 | 107.0 | -170 | -82 | T-CA-d4 | 6.1 |
| T-β-MCA | negative | 514.4 | 124.0 | -175 | -77 | T-CA-d4 | 6.3 |
| T-CA | negative | 514.2 | 107.0 | -155 | -86 | T-CA-d4 | 10.8 |
| T-UDCA | negative | 498.3 | 79.9 | -200 | -124 | T-CA-d4 | 10.0 |
| T-CDCA | negative | 498.3 | 107.1 | -200 | -82 | CA-d4 | 15.1 |
| T-DCA | negative | 498.3 | 79.9 | -170 | -130 | CA-d4 | 16.1 |
| T-LCA | negative | 482.2 | 80.0 | -170 | -126 | DCA-d4 | 20.2 |
| LCA-d4 | negative | 379.2 | 379.2 | -160 | -18 |  | 25.9 |
| T-CA-d4 | negative | 518.4 | 107.0 | -130 | -86 |  | 10.6 |
| DCA-d4 | negative | 395.2 | 395.2 | -145 | -18 |  | 21.4 |
| CA-d4 | negative | 411.3 | 411.3 | -140 | -18 |  | 16.7 |

ESI, electrospray ionization; Q1, precursor ion; Q3 product ion; m/z, mass-to-charge ratio; DP, declustering potential; CE, collision energy; *v*, volt; IS, internal standard; RT, retention time.


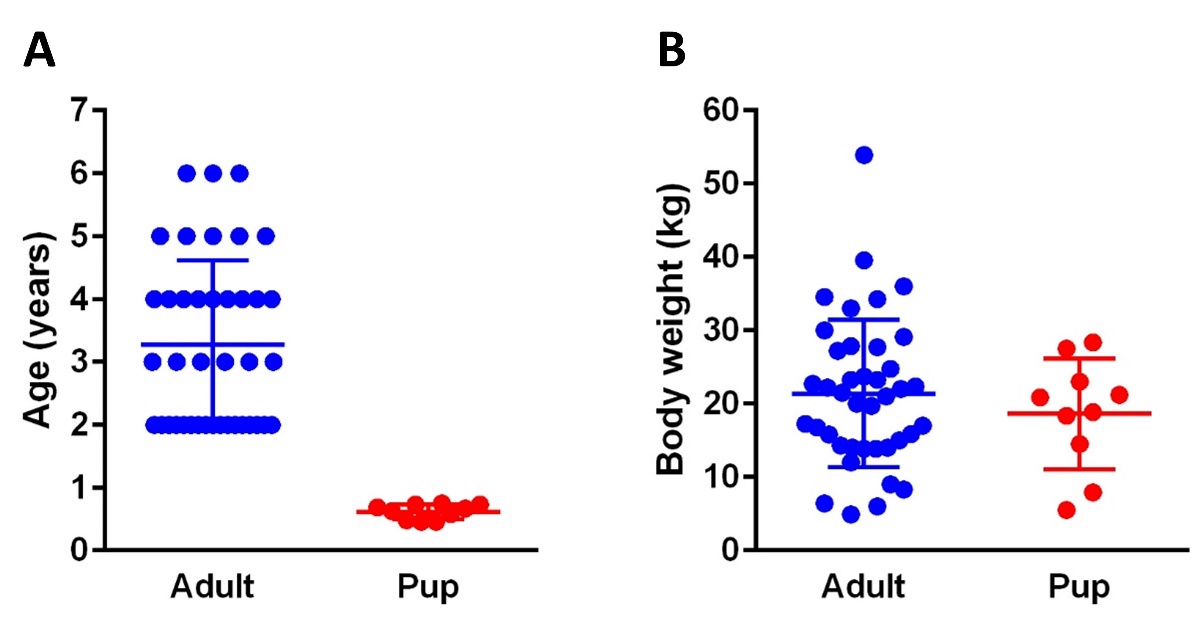
**Supplementary Figure S1.** Age (A) and body weight (B) status of the study population.


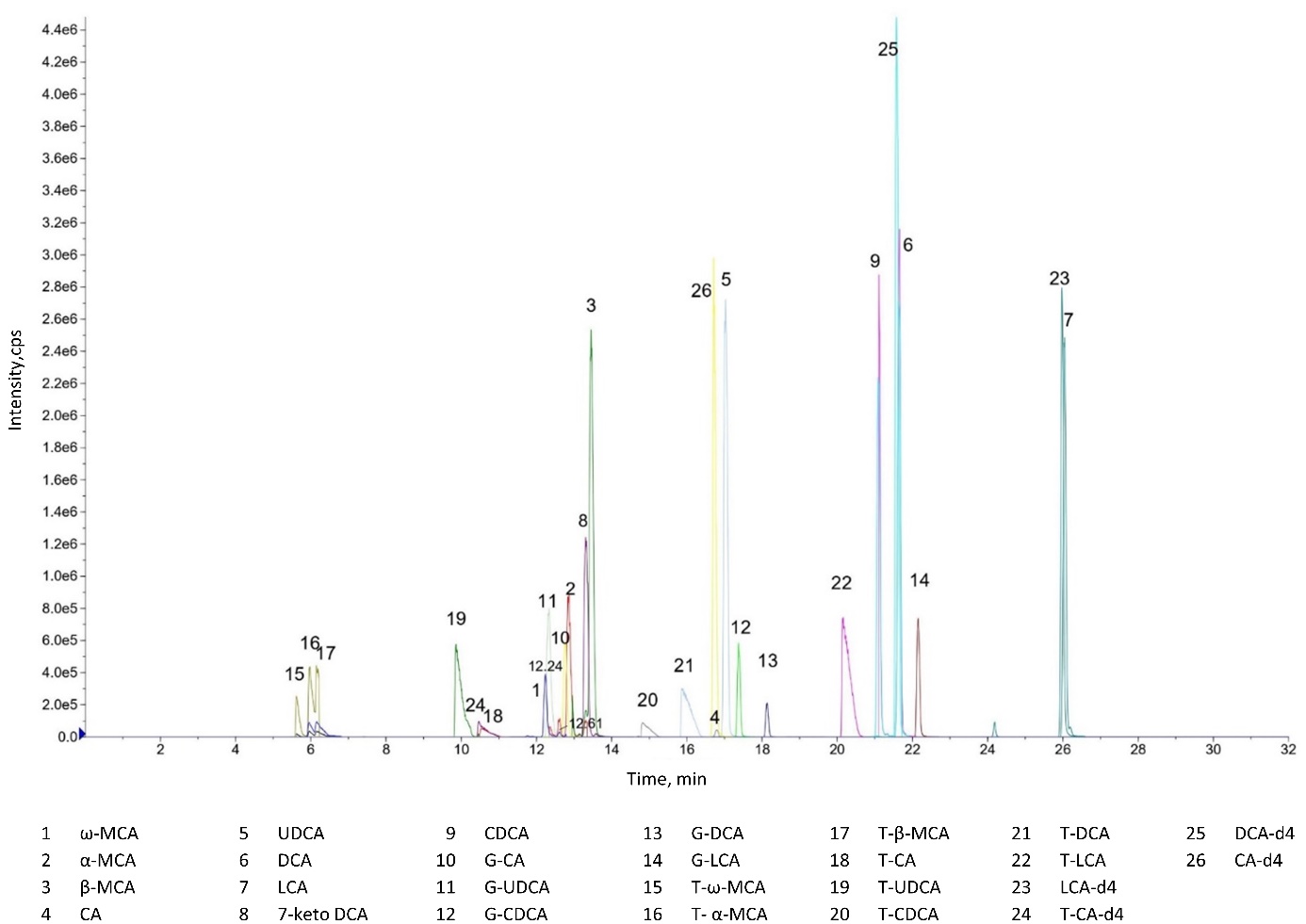


**Supplementary Figure S2.** UHPLC-MRM/MS chromatogram of dog BA.


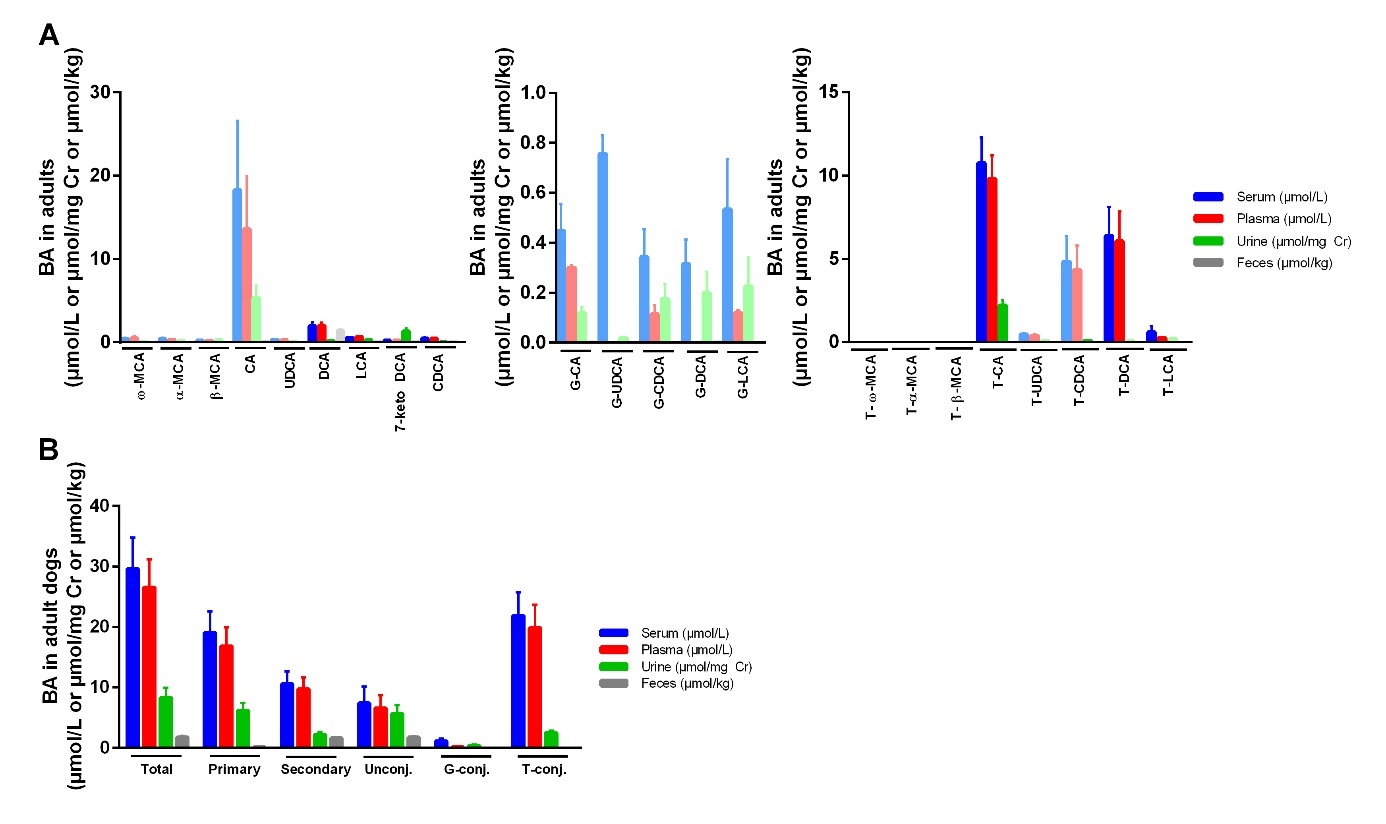


**Supplementary Figure S3. Individual BA concentrations in serum, plasma, urine, and feces of adult dogs.** (**A**) Dark color represents a dataset of n = 40, whereas the datasets n < 40 are indicated by light color. (**B**) BA grouped by type of modification (total, primary, secondary, taurine-, or glycine-conjugated, and unconjugated BA). Data are shown as mean ± SD.


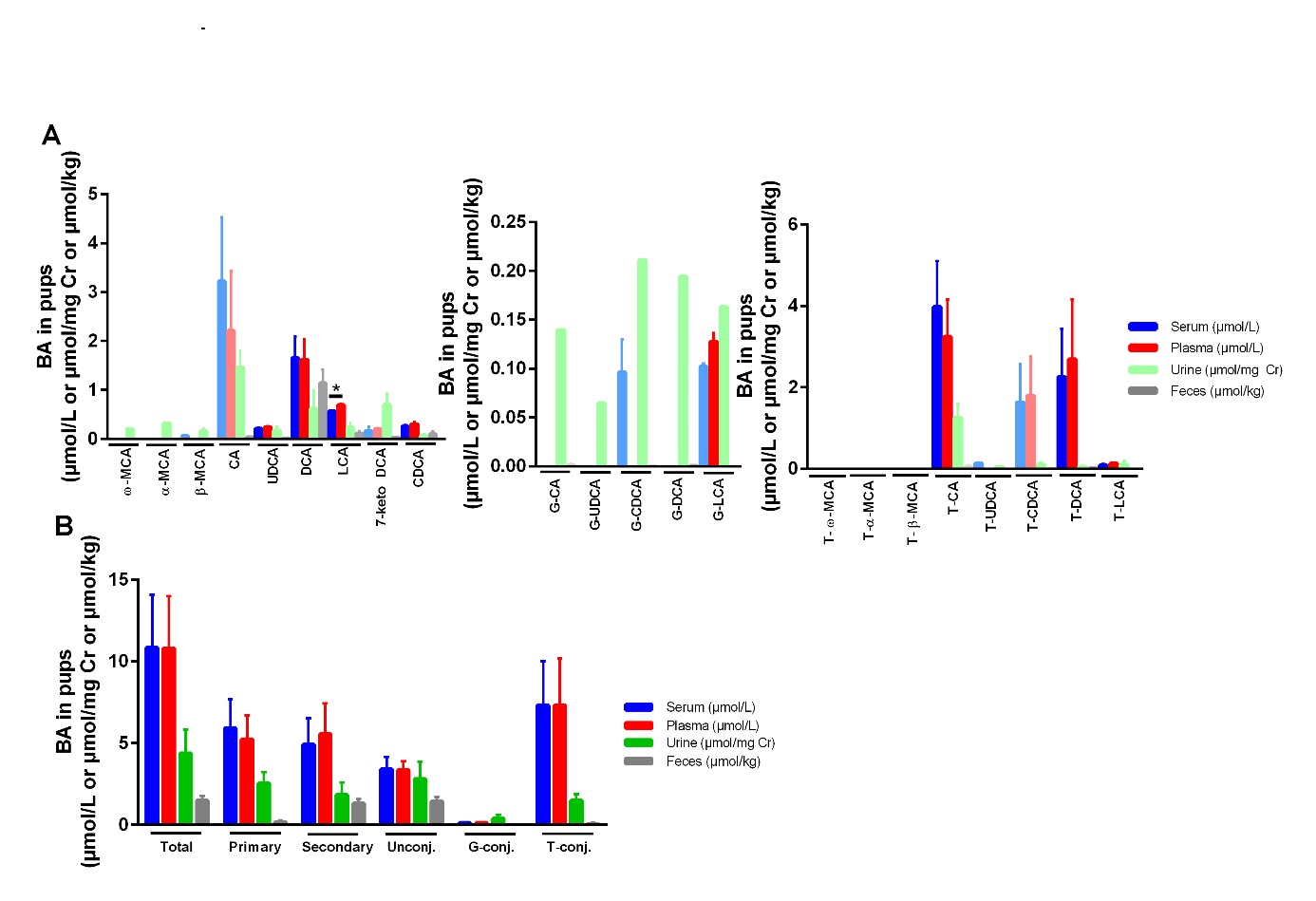


**Supplementary Figure S4. Individual BA concentrations in serum, plasma, urine, and feces of pups.** (**A**) Dark color represents a full dataset of n = 10, whereas datasets with n < 10 are indicated by light color. (**B**) BA grouped by type of modification (total, primary, secondary, taurine-, or glycine-conjugated, unconjugated BA). Data are shown as mean ± SD. Asterisk indicates a statistically significant (*p* < 0.05) difference in the BA concentration between serum and plasma.
